# 

**Published:** 1900-07

**Authors:** 


### BOOKS RECEIVED.

Twenty-fifth Annual Report of the State Board of Health of the State of Michigan. For the fiscal year ending June 30, 1897. Henry B. Baker, M. D., Secretary. Lansing, Michigan. 1900.

Transactions of the Twenty-first Annual Meeting of the American Laryngological Association. Held in the City of Chicago, Ill., May 22, 23 and 24, 1899. New York: D. Appleton & Co. 1900.

A Text-Book of Practical Therapeutics. With Especial Reference to the Application of Remedial Measures to Disease and their Employment upon a Rational Basis. By Hobart Amory Hare, M. D., Professor of Therapeutics and Materia Medica in the Jefferson Medical College of Philadelphia. With special chapters by Drs. G. E. deSchweinitz, Edward Martin and Barton C. Hirst. New (8th) edition. In one 8vo volume of 796 pages, with 37 engravings and 3 colored plates. Cloth, $4.00; leather, $5.00 net. Philadelphia and New York: Lea Brothers & Co. 1900.

Normal Histology. By Edward K. Dunham, M. D., Professor of General Pathology, Bacteriology and Hygiene in the University and Bellevue Hospital Medical College, New York. New (2d) edition. In one very handsome 8vo volume of 319 pages, with 244 illustrations. Cloth, $2.50 net. Philadelphia and New York: Lea Brothers & Co., Publishers. 1900.

A Manual of Obstetrics. By A. F. A. King, M. D., Professor of Obstetrics and Diseases of Women in the Medical Department of the Columbian University, Washington, D. C., and in the University of Vermont, etc. New (8th) edition. In one 12mo volume of 612 pages, with 264 illustrations. Cloth, $2.50 net. Philadelphia and New York: Lea Brothers & Co., Publishers. 1900.

Suggestions to Medical Writers. By George M. Gould, M. D. Duodecimo, pp. 185. Philadelphia: The Philadelphia Medical Publishing Co. 1900.

Medical and Surgical Report of the Presbyterian Hospital, City of New York. Volume IV., January, 1900. Andrew J. McCosh, M. D., and W. Gilman Thompson, M. D., editors.

Atlas and Epitome of Special Pathologic Histology. By Docent Dr. Hermann Duerck, Assistant in the Pathologic Institute; Prosector to the Municipal Hospital L. I. in Munich. Authorised translation from the German. Edited by Ludvig Hektoen, M. D., Professor of Pathology in Rush Medical College, Chicago. Price, $3 00 net. Philadelphia: W. B. Saunders. 1900.

Annual and Analytical Cyclopedia of Practical Medicine. By Charles E. deM. Sajous, M. D., and 100 associate editors. Assisted by corresponding editors, collaborators and correspondents. Illustrated with chromo-lithographs, engravings and maps. Volume V. Methyl-Blue to Rabies. Philadelphia, New York and Chicago: F. A. Davis Co. 1900.

Contributions from the William Pepper Laboratory of Clinical Medicine, University of Pennsylvania. Published on the Phoebe A. Hearst foundation. Edited by Alfred Stengel, M. D., Director. Philadelphia. 1900.

Diseases of the Chest, Throat and Nasal Cavities. Including Physical diagnosis and diseases of the lungs, mediastinum, heart and aorta, laryngology and diseases of the pharynx, larynx, nose, thyroid gland and esophagus. Fourth edition, revised. With 255 illustrations; 8vo, 765 pages. By E. Fletcher Ingals, A. M., M. D., Professor of Laryngology and Practice of Medicine, Rush Medical College; Professor of Diseases of the Throat and Chest, Northwestern University Woman's Medical School, etc., etc. New York: William Wood & Co. 1900. [Price, muslin, $4 50; sheep, $5.25.]

---

### LITERARY NOTES.

The *Journal of Surgical Technology* is the title of a new periodical to be published monthly, beginning July 1, 1900. It will be devoted to the consideration of the technic of surgical procedures, at a subscription price of $1.00 a year. Premiums are offered with the first subscriptions. Address the Technique Publishing Co., 404 East 14th street, New York City, N. Y.

### INDEX TO VOLUME XXXIX.

(New Series.)

PAGE.

"ABDOMINAL CELIOTOMY," 138
Acromegaly . . 192
Acute Dysentery . . . . 132
Albumens, Tests for . . . . . . . 344
Alcohol, Influence of, on Respiration, 598
in Vegetable Kingdom . . 599
Alumni Association Meeting . . . 766
Pen Pictures of . . . . 774
American Medical Association, Journal of, and Medical Society State of New York . . . . 614
American Medical Association, Section on Materia Medica . . . . 858
Anesthetics, Administration of . . 325
Aneurism of the Aorta . . . . . 288
Angell, Edward B., Two Cases of Myelitis . . . . . . . . 661
Antitoxin and Diphtheria . . . . . 779
Antitoxin of Diphtheria . . . . . . 122
Appendicitis, Unusual Conditions Following Operation for . . . . 737
Appendix, The, and the Knife . . . 842
Arecoline, Note on . . . . . . 103
Army Medical Staff, Bill to Increase, 456
Asthmatic, Hygiene of the . . 405
Asylum, Chinese Foundling . 845
Atropine- Use of, in So-called Ophthalmias . . . . . . . 340
Axillary Artery, Suture of . . . . . 191

BACTERIA IN BOOKS . . . . 615
Bath House, First Free Public Municipal, at Buffalo . . 808
Beahan, A. L., Uterine Hemorrhage, 657
Becker, Tracy C., In re Christian Science Ordinance . . . 139
Bissell, W. G., Incineration of Garbage and Excrement at Military Camps . . . . . . 499
Bladder, Accidental Wounds of the Female . . . . . . 448
Books Received . . 75, 154, 237, 314, 395, 475, 553, 634, 714, 797, 869, 942
Buffalo Academy of Medicine, Section on Ophthalmology, Otology, Rhinology and Laryngology Established . . . . . . . 462
Buffalo Enquirer Criticises the Cult, 441
Burns, Cause of Death by . . . . . 186

PAGE.

CANCER, Again the Question of, 559
and Lambert Lack . . 56
Cardiac Inflammations in Children . . . . . . . . 683
Casein, Gastric Digestion of . . . . 598
Cataract, Medicinal Treatment of . . 881
"Celiotomy, Abdominal" . . . 138
Celluloid Sutures and Ligatures . . 187
Cephalagra and Tic-Douloureux from Accessory Sinus Affection . . 423
Children, Experimental Study of . . 193
Cholelithiasis, Indications for Operation in . . . . . . . . . . 191
Cholera Infantum . . . . . . . . 130
Chorea, Etiology and Treatment of . 910
Christian Science and Vaccination . 369
Methods . . . . 1
Ordinance . . . 284
Two Deaths from, 367
Chronic Dyspepsia, Treatment of . . 686
Clayton, Mary, Pathogenesis of Gout, 18
Clemesha, J. C., A Note on Arecoline, 103
Sensory Phenomena in Megrim 488
Clinical Thermometer as a Germ Carrier . . . . . . . 479
Clinton, Marshall, Importance of Early Diagnosis and Surgical Intervention in Fractures of the Skull . . . . . . . . . . . . 79
Clipping Habit, Unacknowledged . . 137
Coakley, J. B., Water Supply and Sewerage Problem of Buffalo . . 568

**College and Hospital Notes:**

Albany Medical College . . . . 936
Batavia Hospital . . . . 304, 936
Buffalo Fresh Air Mission Hospital . . . . 63, 936
Buffalo Hospital Sisters of Charity . . 464, 547, 699
Buffalo State Pathological Laboratory . . . . 65
Charlotte Williams Hospital, Richmond, Va . . . . . . . . 302
Chicago Physiological School . 228
Children's Hospital, New Orleans . . . . . . . . 464

PAGE.
Consumption Pavilion, Erie Co. Hospital . . . . . 699
Emergency Hospital, A New . . 387
Hospital College of Medicine, Louisville . . . . 64
Hospital Ship Maine . . . . . 387
Jefferson Medical College . . 227
Jefferson Medical College Hospital . . . . . . . 785
Lafayette Hospital . . . . . 387
Margaret Fahnestock Training School for Nurses . . 785
Marine Hospital at Buffalo 465
Medical College of Virginia . 864
Medical Department, University of Buffalo . 224, 304, 698,
New Orleans Polyclinic . . . 699
New York Post-Graduate Hospital . . 548
New York Skin and Cancer Hospital . . . . . 305, 388
New York State Hospital for Treatment of Incipient Tuberculosis . . . 787
Niagara Falls Maternity Hospital . . . . . . . . 388
Owensboro, Ky., New City Hospital . . . . 463
Pan-American Hospital . . . 149
Riverside Hospital . . . . 228
St. Vincent's Hospital . . 228, 548
Tuberculosis Hospital for Sailors . . . . . . . . . . 64
Utica State Hospital . . . . 304
Vassar College Infirmary . . 464

Conklin, W. L., Clinical Thermometer as a Germ Carrier . . . 479
Consumption and Christian Science, 439
Plantation Treatment of . . . . . 596
State Control of . . . 602
Consumptives, State Care of . . . 456
Conviction for Illegal Practice of Medicine . . . . . . . . . 456
Cornea, Treatment of Errors in Curvature of . . . . . . . . 761
Corneal Horn . . . . . . . . . 763

**Correspondence:**

Davis and Hahnemann, Henry R. Hopkins . . 607
Substitution and Adulteration, Farbenfabriken of Elberfeld Co . . . . . . . . . . . . 608

Coryza, Acute, Desiccated Suprarenal Capsule in . . . 585
Cott, George F., Radical Operation in Middle-Ear Diseases . . . . . 666

PAGE.
Coulter, C. W., Neurasthenia from Standpoint of General Practitioner, 82
Craig Colony, Report of Managers of . . . . . 455
Cystitis, Curettage of Male Bladder for Chronic . . 825
Tubercular, Treatment of . 844

DAGGETT, B. H., Sterilisation of Catheters, 491
Tests for Albumins . . . . 344
Dangerous People . . . . . 59
Dayton, L. P., In Memoriam . . . . 839
Deafness, Catarrhal . . 14
Death-rate in State of New York . 616
Dennis, Frederic S., at Buffalo Academy of Medicine . . . 690
Diabetes Mellitus . . 40
Doctor with Beliefs, A . . . . . . . 282
Dog Ordinance . . . . 297
Dowd, J. Henry, A New Shut-off . 131
Note on Spermatocele . . . . . . 343
Drugs, Addiction to . . . 909
Dyspepsia, Nervous . . . . . . . . 336

EAR DISEASES, Middle, Radical Operation for 666
Eddy, Mrs., Work of, Analysed, 281
Edema . . 176
Ellis, Charles E., Tracheotomy for Croup . . 269
Elsner, S. L., Presentation of Patient after Kraske's Operation . 485
Epidemic Diseases, To Prevent the Introduction and Spread of . . 779
Epilepsy, Autointoxication in . . . 41
Operations a Failure . . 188
Treatment of, in its Incipiency . . . 799
Experience, Judgment and Luck . 89
Erysipelas . . . 821
Eucaine B., Local Anesthesia with . 841
Eye, Affected by Pathological Conditions of Teeth 366
Relation of Autointoxication and Autoinfection to Diseases of . . . . 399
Eyeball, Complications Following Enucleation of . . . . . . 367
Eyesight in Childhood . . . . . 457

FAITH HEALER'S Idolatry . . 369
Hearing before Councilmen, 379
Ordinance . . 454
Feeble-mindedness, Prevention of . . 683

PAGE.
Fees, Why Not Tap the . . . . . 57
Finerty, John J., Oculist vs. Optician —A Protest . . . 99
Food Products, Adulteration of . 443
Foreign Bodies, Removal of, from Nose and Ear . 601
Fracture of the Hip, Personal Experience with . 829
Fractures of Skull, Importance of Early Diagnosis and Surgical Intervention in . . . . . 79
Fronczak, F. E., Resuscitation of Apparently Dead New-born by Laborde's Method . 418
Frye, Maud J., Manifestations of Lithemia in the Young . . . . 833

GALL-BLADDER, Removal of 913
Garbage and Excrement, Incineration of . . . . 499
Gloves in Surgical Operations 288
Gonorrheal Infection . . . . 122
Gout, Pathogenesis of . . . . 18
Greene, George W., Personal Experience with Fracture of the Hip 829
Grove, B. H., Notes on the Advancement of the Ocular Muscles . 879
Thoughts Suggested by the Local or Medicinal Treatment of Cataract . . . . 881
Guaiacol as an Anesthetic . . . . . 842
Gunshot Wounds, Rest in . . . . 189
Gynecological Mistakes . . . . . . 871

HALL, A. L., Missile and Weapon . . . . . . . . . . 727
Hand Cleanliness . . . . . 687
Harrington, Dr. D. W.'s Aseptic Office . . . 540, 690
Hayd, H. E., Gynecological Mistakes, Their Pathological Bearing and Importance . . . . 871
Pus in the Pelvis . . . 319
Headache, Relation of to Affections of the Eye . 195
Headaches Caused by Eye-strain . . 763
Healers, A Decision Against . . . . 201
Health Department, Records of . . 381
Heath, W. H., Partial Atresia of the Urethra . . 184
The Student in Medicine . . . 239
Hemorrhoids, Treatment of . . . 190
Heroin Hydrochloride . . 685
Himmelsbach, Geo. A., Malaria Complicating the Puerperium . . . . 159

PAGE
Hinkel, Frank Whitehill, Operative Treatment of Sinusitis, 249
Suppuration of Middle-Ear and Empyema of Nasal Accessory Sinuses, Chronic . 746
Hoffman's Anodyne . . . . . 42
Horsehair Scrubber . . . . . . . . . 187
Hospital Liability . . . 127
Hospitals, New, for New York City, 780
Hot Water Drinking . . 449
House-to-House Operating . . 259
Howe, Lucien, The Haab Magnet . 743
Use of Atropine in So-called Ophthalmias . . . . . . 340
Howell, Stephen Y., Edema . . . . 176
Hubbell, A. A., Relation of Autointoxication and Autoinfection to Diseases of the Eye . . . . . 399
Hydatid Cysts of the Liver . . . . 189

**Illustrations:**

Bissell: Incineration of Garbage, etc., at Military Camps.
Fig. 1, Sectional View of Incinerator . . . . 502
Fig. 2, Incinerator Ready for Use . . . . 503
Conklin: Clinical Thermometer as a Germ Carrier. Plate . . 481
Dayton, Lewis P. Plate . . . 860
Dorland, Elias T. Plate . . 618
Hall: Missile and Weapon. Fig. 1, Caliper Micrometer, 728
Fig. 2, plate facing . . . 728
Fig. 3, Vernier Caliper . 731
Harrington: Aseptic Consulting Room . . 691
Howe: The Haab Magnet 744
Lothrop, Thomas. Plate facing 51
Robbins, W. E. Plate . 459
Wende, Ernest: Establishment of Free Public Bath House at Buffalo.
Fig. 1, Simon Baruch, 809
Fig. 2, Goodwin Brown, 811
Fig. 3, Public Bath House No. 1 . 813
Fig. 4, Public Bath Ground Plan 815
Fig. 5, Waiting-room and Office . 817
Fig. 6, Under the Shower . . . 819
Wende, Ernest and G. W.: Rhinophyma: Fig. 1, facing . . . 207
Fig. 2 . . . . . . . 210

PAGE.

INFANT MORTALITY, Cause and Prevention of . . . 446
Infectious Diseases, Acute, Excretion in the Treatment of . 600
Indiana Opinion, An . . . . . . . 280
Ingersoll, J. M., Causes and Effects of Mouth Breathing . . . 414
Inguinal Hernia in Children . . . 121
Insane, Physical Condition of . . . 197
Insanity, Thyroid Extract in . . . . 759
Intestinal Suture, A Point in . . . . 191
Involution, A Study in . . . . . . 197
Iodine, Action of . . . . . 597
Iodoform, To Remove Odor of . . 103
Iowa, License to Practise in . . . . 616

**Instruments, New :**

A New Shut-off . . . . . . . . 131

**Items :**

Appleton, D. & Company . . . 717
Arlington Chemical Co. . . . . 718
Brigham Hall . . . . . . 157
Christian Science Scoffer . . . 317
Delegates to Medical Press Congress at Paris . . . . . . . 798
Fairchild Bros. & Foster's Desk Diary . . . . . . . . . 638
Faith Healers Indicted . . . . 318
Fellows, James I . . . . 718
New York Pharmacal Association Calendar . . 944
Of Interest to Physicians . . . 944
Palisade Manufacturing Co . . 718
Paris and the Midnight Sun . . 798
Paris Exposition Excursion . . 638
Pen Carbon Letter Book . . 718
Wyeth, John & Bro . . . . 718
Sander Prize . . . 637
Saunders, W. B. & Company . 870

JACK, GEORGE N., Hygiene of the Asthmatic . . . . . 405

KENTUCKY State Board of Health, Requirements of . . 138
Kernig's Sign . . . . . . . 196
Kraske's Operation and Presentation of Patient . 485
Krauss, William C., Medico-Legal Aspects of Trauma, etc . 164
Taking Inventory of Nervous Case . . . . . . . 890
Kidney, Granular . . . . 44
King, Clarence, Fad, Fact and Fancy in Medicine . . . . . . . . . . 576

PAGE.

LABORDE'S METHOD in Resuscitation of the New-born, 418
Lacto-Somatose . . . . . . 50
La Grippe . . . . . . . 204
Laryngitis, Diabetic . . . . . . . . 846

**Leading Articles :**

Acting Assistant Surgeons U. S. Army . . . . . . . . . 454
A. D. 1900 . . . . . 450
Alumni Banquet . . . . . . . 852
Alumni Reunion . . . 612
American Medical Association at Atlantic City . . . 928
Antivivisection Redivivus . 536
Bennett Park and the New Emergency Hospital . 374
Board of Examiners in Midwifery . . . . 857
Christian Science Deception . . 53
Christian Science Ordinance 136
Consumption, Out-door Treatment of in Pennsylvania 930
English and American Contract Surgeons 452
False Reports Credited as Military News . . . . . 292
Giving of Death Certificates . . 216
Head or Figurehead . . . . . 217
Honors for Dr. Jacobi . . . 777
How Michigan Defines the Practice of Medicine . 293
Hydrophobia "Epidemic," The . 133
International Medical Congress of 1900 . . . 377
Lapidotherapy . . . . . . 538
Lothrop, Dr.,—Volume LV. . . 51
Medical Department Grosvenor Library . 856
Medical Directorship of the Exposition . . . . . 212
Medical Legislation at Albany . 688
Medical Society State of New York . . . 609
Mortality Statistics in the Twelfth Census . . . 296
New Medical Library Building . 848
New Medical Organisation in Erie County . 847
Newspapers, Medicine and Charity . . . . 375
Osteopaths Must Leave Kentucky . . . . . . 453
Overcrowding in Nursing Profession—A Remedy . . . . 134
Professional Education . . . . 532
Sanitation at the Fair . . . . 215
Street Noises and Health . . . 213
Sweeping Train . . . . . . . 535

PAGE.
Therapeutic Need, A . . . . . 54
Units of Remedial Agencies . . 372
University Day . . . . . . . . 765

**Literary Notes:**

American Medical Association Prize Medal . . . . . . . 555
Anglo-Saxon Review . . . . . 715
Annual Report Children's Hospital of Buffalo . . . . . 635
Antikamnia Skeleton Calendar 476
Archives of Neurology . . . . 477
Archives of Pediatrics . . . . 555
Battle & Co.'s Blood Dyscrasia . 478
Bibliographia Medica . . . . . 717
Bookman, The . . . . . . 715
Breitenbach Co.'s Year Book . . 477
Bulletin of Cleveland General Hospital . . . . . . . 75
Coca, Its Therapeutic Application . . . . . . . . . . . . 475
Columbia Desk Calendar . . . 478
Columbus Medical Journal . . 717
Deaver's Human Anatomy . . 556
Doctor's Magazine . . . . . . 636
Hippocratic Oath by Arlington Chemical Company . . 77
Illinois Medical Journal . . 157
Journal of Surgical Technology, 943
Lea's Pocket Text-Books . . 557
Louisville Monthly Journal of Surgery and Medicine . . . 76
Maryland Medical Journal . . . 555
Mechanics of Surgery . . 637
Medical Libraries . . . . 77
Mellier Drug Company's Old Engraving . . . . 78
Memphis Medical Monthly . . 944
New York Lancet . . . . . 635
North Carolina Medical Journal, 555
Occidental Medical Times . . . 156
Providence Medical Journal . . 555
Quarterly Medical Journal . . . 156
Regular Medical Visitor . . . 636
Samuel D. Gross Prize . . . . 716
Saunders's American Year Book of Medicine and Surgery . . 556
St. Louis Courier of Medicine . 156
Stylus, The . . . . . . . . . 636
Texas Clinic . . . . . . 156
Texas Medical News . . . . 238
The Iris . . . . . . 556
Treat's International Medical Annual . . . . . . 557
University Medical Magazine . 717
Vaccine, Propagation of . . . 398
Vermont Medical Monthly . 316
Warner's Pocket Medical Dictionary . . . . . . . . 156

PAGE.
W. B. Saunders's Announcement of New Books . . . . 76
W. R. Warner & Co.'s List . . 77
Wm. R. Warner & Co.'s Silver Medal . . . . . . . . 478
Wilkinson's Medical Digest . . 75
William Wood & Co's Limited Editions . . . 636
William Wood & Company's Reference Handbook of Medical Sciences . . . . . 944
Yellow Fever Reports, U. S. Marine Hospital Service . . 316

Le Breton, Prescott, Subphrenic Abscess . . . . . . . . . 255
Legrand's Solution . . 841
Leprosy, Law for the Prevention of . 692
Ligation of Uterine Arteries . . . 130
Ligature, Slipping of Intraperitoneal, 24
Lithemia, Manifestations of, in the Young . . . . . . . 833
Loud Bill Again . . . . . . . . . 693
Loveland, B. C., Nervous Dyspepsia, 336

MAGNET, Form of the Haab . 743
Major-General Editors . . 381
Malaria Complicating the Puerperium . . . . 159
Pen Pictures of . . . 265
Maltine with Creosote . . . . . . . 46
McGuire, Frank W., The Administration of Anesthetics . . . 325
Medical Association County of New York no Longer Independent . . . . . . 858
Association of Central New York . . . . 902
Examiners' Report . . . . 59
Practice in Cuba . . . . 130
Students of Ohio Lobbying, 616
Medicine, Fad, Fact and Fancy in . 576
The Student in . . 239
Medico-Legal Aspects of Trauma in Relation to Diseased Cerebral Arteries . 164
Department at Manila, 397
Megrim, Sensory Phenomena in . 488
Mental Derangement and Renal Disease . . . . 760
Diseases, Classification of . . 192
Metrorrhagia and the Menopause . . 885
Michigan, A Medical Law for . . 203
Middle-Ear, Chronic Suppuration of, 746
Midwifery Bill . . . . . . . . . 614
Millener, Frederick H., Desiccated Suprarenal Capsule in Acute Coryza . . . . . . . 585
Minimum Standards for Admission to Practise . . . . . . . . . . 93

PAGE.

**Miscellany :**

A Good Practice for Sale . . . 78
Antikamnia Company's Pocket Folder . . . . . . . 316
California Fig Syrup Co.'s Injunction vs. Worden & Co . 78
Civil Service Examination . . 158
Native Drug Plants of U. S . . 557
Osteopathy in Georgia . . 558
Physician's Diploma for Sale . . 558
Physician's Pocket Manual . . 398
Practising Medicine Illegally . . 558
Relief for Porto Rico . 158
Scott & Bowne's Desk Calendar, 558
William F. Jenks's Memorial Prize . . . . . . . . . . . 78

Missile and Weapon . . . 727
Mooney, E. L., Duty of the General Practitioner Toward the Specialist, 263
Mouth-Breathing, Causes and Effects of . . . . 414
Myelitis, Two Cases of . . . 661
Myopia, Suppression of Crystalline Lens in . . . 436

NASAL Accessory Sinuses, Empyema of . . 746
Atresia, Congenital . . 206
Needle, To Thread, Religiously . 842
Needles, To Preserve . 842
Nervous Case, Taking Inventory of . 890
Diseases, Effects of Season in Causing 890
Prostration, Headache and Neuralgia, Treatment of . 648, 719
System in Pulmonary Phthisis 911
Neurasthenia . . . 82
New Hampshire, Consumption on Decrease in . 616
New York State Medical Association, Charter of 692
Noise Nuisances . . 297
Nose, To Extract Foreign Bodies from . . . . . . 843
Nuclein in Septicemia . . . . . 42
Nurses Wanted in Cuba . . . . . 780

OCULAR MUSCLES, Notes on Their Advancement . 879
Oculist vs. Optician . . 99
Operative Procedure, Essential Requirements of . . . . . . 287
Orthoform, Clinical and Experimental Use of . . . 914
Osteopathy, Illegal in Pennsylvania . 455

PAGE.

Otitis Media in Infancy . . . . 681
Oxyuris Vermicularis . . . . . 40

**Obituary :**

Appleton, Wm. H. . . . . . . . 299
Axtell, E. R . . . . . 542
Bourne, Mrs. C. W . . . . . . 385
Canfield, Wm. B . . . . . . . 542
Charpentier, A. L. A. . . . . . 143
Cleary, John C . . . . . . 544
Dayton, Lewis P . . . . . . 859
Dorland, Elias T . . . . . . . 617
Eddy, Loren L . . . . . . . 696
Gibier, Paul . . . 933
Graham, James Elliott . . . . 142
Gray, Landon Carter . . . 861
Hammond, William A. . . 544
Hunt, Martha T. . . . . . 617
Jackson, Thomas . . . 543
Lattin, George . . . . . 782
Merrick, Myra K . . . . . . 385
Miller, William O. . . . . 386
Mudd, Henry H . . . . . 460
Mulheron, Edward . . . . . 782
Otis, Fessenden N. . . . . 861
Paget, Sir James . . . . . 543
Palmer, George S . . . . . . . 782
Pardee, Charles Inslee. . 385
Park, Mrs. Roswell . . . . 384
Priestly, Sir William O. . . . 783
Robbins, W. E . . . 458
Smith, Clifton G . . . . . . 299
Spencer, H. G. P. . . 141
Stanbro, Edward Everett . . 384
Stewart, Sir Thomas Grainger 696
Storrs, Melancthon . . . . . 934
Sutherland, Joseph H . . . . 932
Volker, Andrew J. . . . . . 460
Whittaker, James T. . . . . . 933
Wright, A. R. . . . . . 695

PARALYSIS of the Heart and the Healers . . . 440
Paris Exposition, Admission to for Members of the Medical Congress . . . 858
Paris, Health of . . . . . . . . 693
Paresis, Etiology . . . . . . . 912
Paretic Dementia . 913
Park, Roswell, Again the Question of Cancer . . 559
Pathology, Section on, in American Medical Association . . . 689
Penitentiary, Report of Physician of, 690
Peptomangan—Gude . . . 47
—Gude, Demonstration of the Use of 843
Peritoneum, New Way of Closing . 188

PAGE.

**Personal :**

Bissell, William G.; Frye, Maud J.; Meyer, Edward J ; Stivers, C. G.; St. John, Charles T. 383
Breuer, Max C.; Sänger, Max; Wall, Charles A. . 141
Briggs, A. H.; Kerr, Abram Tucker; Hartwig, Marcel; Gant, Samuel G. 931
Bruce, Hortense V.; Stockton, Charles G.; Lewis, F. Park . 694
Brush, Edward N.; Woodbury, John McG.; Mathews, Joseph M.; McMurtry, L. S.; Love, I. N . . . 60
Burr, C. B.; Ill, Edward J.; St. John, Charles T.; Doty, A H., 541
Cameron, John W.; Palmer, George S. . 616
Chapin, Edward; Wasdin, Eugene 782
Carpenter, T. B.; Cameron, John W.; Noble, Charles P.; Jewett, Carlton R.; Wilson, Nelson W.; Mickle, Herbert; Meyer, Edward J.; Mynter, Herman; Hubbell, A. A. . . 219
Dorsett, Walter Blackburn 59
Fronczak, Francis E.; Howard, Charles F . . . 781
Heard, Thos. M., Jr.; Shaw, O. C.; Loveland, B. C. 542
Hinkel, Frank Whitehill; Hall, Rufus Bartlett 61
McGuire, F. W.; Mills, F. H.; Bingham, F. P.; Lothrop, Earl P . . . . . . . . 458
McGuire, F W.; Jacobi, A.; Smith, Eugene A.; Goldberg, S.; Russell, N. G.; Wende, Ernest . . . . . 932
McMurtry, L. S.; Stivers, C. G., 139
Mynter, Herman; Martin, A ; Mulford, Henry J.; Krauss, Dr. and Mrs. William C.; Crego, Floyd S.; Thompson, F. D . . . . . 140
O'Brien, E. C. W.; Smith, E. T., 398
Park, Roswell; Potter, Julius H., 695
Park, Roswell; Long, Eli; Hoag, Myrtle A.; Ernest, J. Glenn; Trowbridge, Grosvenor R.; Nichell, Henry . . . . 859
Putnam, James Wright; House, Wm.; Robison, Joseph H.; Kerr, Abram T. . . . . . . 298
Wood, Leonard . . . . . . . 457

Peroxide of Hydrogen in Removal of Dressings . . . . . . . . . . 927

PAGE.

Pertussis . . . . . . . . . . . . . 43
Treatment of . . . 449
Peterson, Frederick, Practical Psychology . . . 639
Philippines, Need of Medical Officers in . . . . . . . 615
Plague Ended at Honolulu . . . . 780
Pneumonia, Hydrotherapy in . . 682
Pollution of Water Supply of Cities, 540
Potter, William Warren, Address of at Alumni Banquet . 770
Metrorrhagia and the Menopause . 885
What Shall be Minimum Standard of Requirements for Admission to Study and Practice of Medicine? . . . 93
Poultice, The Good of . 840
Practice of Medicine and Christian Science . . . . . . . 58
Practitioner, General, Duty of, Toward the Specialist . . . . . . . 263

**Progress in Medical Science :**

Genito-Urinary and Syphilitic Diseases, by B. H. Daggett 44, 122, 276, 444, 600
Illegal Medicine, by Nelson W. Wilson . 279, 367, 439
Legal and State Medicine, by Wm. H. Heath 127, 602
Medicine, Pathology and Therapeutics, by Albert T. Lytle . 41
Neurology, by Wm. C. Krauss 192, 759, 909
Obstetrics, and Gynecology, by William Warren Potter . 285, 905
Ophthalmology, by Alvin A. Hubbell 366, 436, 761
Pediatrics, by Maud J. Frye 40, 121, 681
Physiological Chemistry, by Jno. A. Miller . . . 597
Surgery, by John Parmenter and Nelson W. Wilson . 186, 287, 840

Prostate, Observations on . . . . . 126
Prostatectomy, Notes on . . . . . 895
Prostatitis, Prescription for . . . 449
Protargol in Gonorrhea . . . . . . 279
Psychology, Practical . . . . . 639
Puerperal Infection, Early Symptoms of . . . . . . . . . 906
Pus in the Pelvis . . . . . . . . . . 319

RANNEY, AMBROSE L., Scientific Treatment of Nervous Prostration, Headache and Neuralgia . . . . . . 648, 719

PAGE.
Reed, Charles A. L., Speech of, at Alumni Banquet . . 854
Reed, Memorial Address on Lawson Tait . . . 218
Regents, Annual Meeting of and Appointments by . . . . 613
Renal Casts . . . . . . . . 444

### Reviews:

Abbott: Hygiene of Transmissible Diseases . . . . . . 229
Abrams: Scattered Leaves from Physician's Diary . . . 940
Allen-Sobel: Handy Book of Medical Progress . 305
Anders: Practice of Medicine . 630
Attfield: Chemistry 152
Barker: Nervous System and Neurones . 468
Bartholow: Materia Medica and Therapeutics . . . 708
Blakiston: Physician's Visiting List . . . . . . . . 394
Bradford-Lovett: Orthopedic Surgery . . . 706
Brubaker: Compend of Human Physiology . . . . . . 236
Bruce: Materia Medica and Therapeutics . 311
Principles of Treatment 937
Bryant: Operative Surgery 795
Butler: Love and its Affinities . 629
Materia Medica, Therapeutics, etc 633
Cabot: Serum Diagnosis of Disease . . 152
Chapman: Human Physiology . 151
Cheyne-Burghard: Manual of Surgical Treatment, Vol. I., 65; Vol. II. . . 627
Church-Peterson: Nervous and Mental Diseases . 73
Coakley: Diseases of Nose and Throat . . . . . 551
Coblentz: Newer Remedies . . 313
Collins-Gallaudet: Physiology . 632
Corwin: Physical Diagnosis of the Thorax 633
Crockett: Diseases of Women . 791
Crook: Mineral Waters of the United States . . 232
Cushny: Text-Book of Pharmacology and Therapeutics . . . 309
Daland: International Clinics, Ninth Series, Vol. I., 71; Vol. II, 308; Vol. III . . 474
Daniel: Recollections and Sketches . . . . . . 795
Dorland: Pocket Medical Dictionary . . . . . . . . . . 475

PAGE.
Dudley: Treatise on Gynecology, 704
Duncan: Children, Acid and Alkaline 793
Eckley: Practical Anatomy . 941
Fuchs: Text-Book of Ophthalmology . . . . 67
Fullerton: Surgical Nursing . . 309
Gillespie: Modern Gastric Methods . . . . 793
Gould-Pyle: Compend of Diseases of the Eye . . 474
Cyclopedia of Medicine and Surgery . . . . 865
Gould: American Year Book . 711
Pocket Dictionary . 942
Gowers: Diseases of the Nervous System, Vol. 1 . 553
Grafstrom: Text-Book of Mechano-Therapy 306
Haab: Atlas of Diseases of the Eye . . . . 74
Hall: Text-Book of Physiology, 470
Hare: Complications, Accidents and Sequelæ of Typhoid Fever . . . . . . 392
Practical Diagnosis (fourth edition) . . . . . 310
Progressive Medicine, Vol. II., 70; Vol. III., 471; Vol. IV., 1899, 550; Vol. I., 1900, 867; Vol. II., 1900 . . . . . . . 940
Principles of Bacteriology 149
Heineck: General and Local Anesthesia . . 631
Heisler: Text-Book of Embryology . . 706
Helferich: Atlas Fractures and Dislocation . . . . . . 314
Hirt: Diseases of the Nervous System . . . . . . . . 236
Holden: Human Osteology 68
Holland: Urine, Gastric Contents, etc. 474
Hollopeter: Hay Fever . 69
Hughes: Practice of Medicine . 552
Hyde: Diseases of the Skin . 792
Index-Catalogue Library Surgeon-General's Office, U. S. Army Second Series, Vol. IV. D—Emulsions . 313
Jackson: Diseases of the Eye 790
Keen-White: American Text-Book of Surgery . 705
Kirke: Handbook of Physiology, 472
Kyle: Diseases of the Nose and Throat . . . . . . . . . . 467
Lea: Medical News Visiting List . . . . . . 394
Letchworth: Care and Treatment of Epileptics . . . . . 549

PAGE.
Lydston: Diseases of Genito-Urinary Tract 623
Malsbary: Practice of Medicine, 709
Martin, Essentials of Surgery, 7th edition . . . . 941
Medical News Formulary, 1899 . 153
Mracek: Atlas of Diseases of the Skin . . . . . . 234
Musser: Medical Diagnosis . . 702
Nichols: Histology and Pathology . . . . 707
Norris-Oliver: Diseases of the Eye, Vol. IV . . 620
Ostrom: Massage and Swedish Movements . . . 308
Pantaloni: Chirurgie du Foie et des Voies Biliaires . 865
Park: Bacteriology in Medicine and Surgery . 703
Treatise on Surgery . . . . 389
Penrose: Diseases of Women . 868
Prescriptions and Favorite Formulæ . . . . . . 233
Pryor: Pelvic Inflammations . . 551
Purrington: Christian Science . 868
Recent Legal Decisions 150
Report of Commissioner of Education, 1896-1897 70
Johns Hopkins Hospital, Vol. VII . . 628
Methodist-Episcopal Hospital, Vol. I., '87-'97 . . 235
Mt. Sinai Hospital, Vol. I., 1898 . . 231
of New York State Board of Health . . . . 713
of State Commission in Lunacy . . . 710
Public Health, U. S., Marine Hospital Service, Vol. XIII. . . 393
Supervising Surgeon-General Marine Hospital Service . 393
Roberts: Principles and Practice of Modern Surgery 939
Rockwood: Physiological Chemistry . . . 634
Roosa: Defective Eyesight . 72
Sajous: Annual Cyclopedia of Practical Medicine, Vol. IV. . 472
Savill: Lectures on Neurasthenia . . . 700
Schleif: Manual of Materia Medica, etc 475
Shattuck: Pathogenic Bacteria, 632
Skene: Electro-Hemostasis in Operative Surgery . . . . . 371
Stedman: Twentieth Century Practice, Vol. XVI., 71; Vol. XVIII., 473; Vol. XIX . . . 861

PAGE.
Stelwagon: Diseases of the Skin, 794
Stevens: Practice of Medicine, 794
Stoney: Materia Medica for Nurses . . . . . . . 154
Summers: Modern Treatment of Wounds . . . . 623
Thorington: Refraction and How to Refract . . . . . . . . 796
Transactions:
American Association of Obstetricians and Gynecologists, 1898. . . 69
American Dermatological Association . . . 711
American Laryngological Association, 1898 . 314
American Medico-Psychological Association 153
American Surgical Association . 796
Medical Association of Central New York 713
Medical Society of the State of North Carolina 69
Medical Society State of New York . 312
Mississippi Valley Medical Association . 866
Ohio State Medical Society, 712
Rhode Island State Medical Society 711
Southern Surgical and Gynecological Association 149
Texas State Medical Association . . . . 713
West Virginia State Medical Society . . . . . 712
Wisconsin State Medical Society . . 711
Wyoming State Medical Society . . . . . . 712
Truax: Mechanics of Surgery . 391
Tuttle: Diseases of Children . 631
Voswinkel: Surgical Nursing . 153
Walker: Introduction to Dermatology . . . . . . 306
Warner: Pocket Medical Dictionary . . . . 634
Warren-Gould: International Text-Book of Surgery Vol. I., 626; Vol. II . . . . . . . . 787
Watson; Handbook for Nurses, 942
Wells: Compend of Gynecology, 471
Wharton: Minor Surgery and Bandaging . . . . . . . . . 552
Wilson: Fever Nursing . . . . 154
Wolff: Medical Chemistry . . 794
Ziegler: General Pathology 465

Rheumatic Conditions . . . . . . 211
Rheumatism, Acute . . . . . . 531

PAGE.
Rhinophyma . . . . 207
Ricketts, Edwin, House-to-House Operating . . . 259
Rohé, George H., Memorial Tablet to . . . . . . . . . . . . . . . 455

SALT SOLUTION, Use of . . . 841
Scarlet Fever . . . 43
School Break-downs . . . 910
Sinusitis, Operative Treatment of Frontal and Maxillary . . . 249
Soble, Nathan W., Curettage of Male Bladder for Chronic Cystitis, 825

**Society Meetings:**

American Academy of Medicine . . . . 696
American Association of Obstetricians and Gynecologists . . . 61, 223
American Electrotherapeutic Association . 63, 143, 934
American Medico-Psychological Association . . . 619
American Microscopical Society, 220
American Proctogical Society 62
American Public Health Association . . . 301
Association of Military Surgeons 148, 223
Buffalo Academy of Medicine . . . 222, 302, 386, 461, 546, 619, 697, 783, 935
Congress Against Tuberculosis . 698
Esculapian Club 224, 386
International Congress at Paris 147
International Congress of Medical Electrology and Radiology, 620
International Medical Congress, Thirteenth . . 460
Lake Erie Medical Society, 222, 460, 934
Lehigh Valley Railroad Surgeons . . 143
Medical Association of Central New York . . 220, 301
Medical Society County of Chautauqua . . 147, 934
Medical Society, County of Erie . . . . . . . 462, 544
Medical Society of Virginia . 301
Medical Society State of New York . . 461, 545
Medical Union of Buffalo . 619
Medico-Legal Society of New York . 547
Mississippi Valley Medical Association . . . . . . . . . . 300

PAGE.
National Confederation of State Medical Examining Boards . 785, 862
National Pure Food and Drug Congress . . 547
New York Academy of Medicine, 546
New York Physicians' Mutual Aid Association 546
New York State Medical Association, 299; Fourth District Branch of . . . . 783
Niagara Falls Academy of Medicine . . . 63, 148, 222, 386, 460, 545, 620, 785
Physician's Club . 547, 863
Rochester Academy of Medicine . . . . . . 935
Southern Surgical and Gynecological Association . . . 300, 462
Surgeons' Association, W. N. Y. & P. R. R 301
Wyoming State Medical Society, 221

**Society Proceedings:**

American Association of Obstetricians and Gynecologists 2 7
Buffalo Academy of Medicine . 39, 144, 271, 587, 750, 837, 893
Section on Obstetrics and Gynecology, 360, 432, 515, 751
Section on Medicine . 362 427, 529, 678, 757, 837, 902
Section on Pathology . . 363, 431, 523, 671, 900
Section on Surgery . . . . 517, 590, 673, 895
Section on Ophthalmology, Otology, Rhinology and Laryngology . . . 526, 677, 754, 898
Medical Association of Central New York 353, 902
Medical Society of the County of Chautauqua . . . 434
Medical Society of the County of Erie 508, 839
National Confederation of State Medical Examining Boards . . 25

Snow, Sargent F., Catarrhal Deafness . 14
Cephalagra and Tic-Douloureux from Accessory Sinus Affection . . . . 423
Sodium Fluoride, Toxic Action of . . 599
Spermatocele, Note on . . 343
Spratling, William P., Treatment of Epilepsy in its Incipiency . . . 799
Standards, Minimum, for Admission to Medical Colleges . . . . . . . . 25

PAGE.

State Board of Medical Examiners, Report of . . . . . . . 615
Sterilisation of Catheters . . . . 491
Sterility, Causes of 286
Stockton, Charles G., Speech of, at Alumni Banquet . . . . . . 852
Streptosyphilis . . . . . . 764
Subphrenic Abscess . . . . . . . . 255
Substitution, the Question of . . . . 380
Sugar, Substitute for in Diabetes . . 206
Sugars, Toxic Action of . 598
Suprarenal Extract, Administration of, by Mouth . . . . . . . 597
Surgeon-General's Annual Report . . 382
Sutton, R. Stansbury, Slipping of the Intraperitoneal Ligature . . . . 24
Syphilis, Insanities . . . . . . . . 123

TAPE WORM . . . 43
Taylor, W. G., Unusual Conditions Following Operation for Appendicitis . . 737
Tenement-House Commission . . . 778
Therapy, History of Opo- or Organo-, 104
Thorndike, Paul, Notes on Prostatectomy . . . . . 895
Toothache Healers and the U. of B., 441
Prescription for . 606
Toxalbumin from the Common Eel . 597
Tracheotomy for Croup . 269
Tumors, Chemistry of malignant . . 598
Typhoid Fever, Diet in 530
Suppression of in Berlin 455
Surgical Complications of . . 114
The Bath in . . 684

URETHRA, Partial Atresia of . . 184
Urine, Detection of Albumin in . . . . . . . . . . . 600

PAGE.

Urinary Toxicity in Pregnant Women, 285
Urine, Incontinence of . . . . . 487
Pentoses in . . . . . . 599
Segregator . . . 125
Uterine Cancer, Early Diagnosis of . 907
Inertia . . . . . . . . 905
Hemorrhage . . . . . 657
Uterus, Cancer of . . . . . . 908
Can Ergot Cause Rupture of, 905

VERMIN, Bites of . . 43
Violators of Medical Laws, Arrest of . 694
Vital Statistics of American Cities . 379
Vulvæ, Pruritus . . . . . . 9c6

WARBURG'S TINCTURE . 41
Water Supply and Disposal of Sewage . 539
Water Supply and Sewerage Problem of Buffalo . . . . 568
Pollution of in Cities . 540
Wende, Ernest, Establishment of First Free Municipal Bath House at Buffalo . . . 808
Ernest and G. W., Rhinophyma . . 207
Wilson, Nelson W., Christian Science Methods . . 1
Experience, Judgment and Luck . . 89
Pen Pictures of Malaria . 265
Witchcraft at the Center . . . . . 442

YELLOW FEVER Serum at Havana . . . . . . . 378
Young, A. A., Erysipelas . . . 821

